# Virus load and clinical features during the acute phase of Chikungunya infection in children

**DOI:** 10.1371/journal.pone.0211036

**Published:** 2019-02-01

**Authors:** Siva Raghavendhar B., Ashok Kumar Patel, Sushil Kumar Kabra, Rakesh Lodha, Vinod H. Ratageri, Pratima Ray

**Affiliations:** 1 Kusuma School of Biological Sciences, Indian Institute of Technology, Hauz Khas, New Delhi, India; 2 Dept. of Pediatrics, All India Institute of Medical Sciences, Ansari nagar, New Delhi, India; 3 Dept. of Pediatrics, Karnataka institute of Medical Sciences, Hubli, India; 4 Department of Biotechnology, Faculty of Science, Jamia Hamdard, Hamdard Nagar, New Delhi, India; CEA, FRANCE

## Abstract

**Background:**

Chikungunya virus (CHIKV) infection is a long known mosquito-borne disease that is associated with severe morbidity, characterized by fever, headache, rashes, joint pain, and myalgia. It is believed that virus load has relation with severity of clinical features.

**Objectives:**

We performed this study to assess the relationship between virus load and clinical features in children during the acute phase of CHIKV infection, in order to draw insights for better-informed treatment.

**Study design:**

Between June 1, 2009, and May 31, 2010, 338 patients with fever and susceptive to CHIKV during first 4 days of illness were prospectively enrolled from Karnataka Institute of Medical Sciences, Hubli in our hospital based cross sectional observational study. Sybr green quantitative reverse transcription polymerase chain reaction was performed to estimate the virus load.

**Results:**

Quantitative RT-PCR was positive for CHIKV in 54 patients. The median copy number of CHIKV was 1.3x 10^8^ copies/ml (1.7x10^5^-9.9x10^9^ copies/ml). Among the observed clinical features, a statistically significant difference in log mean virus load was found between patients with and without myalgia (log mean 7.50 vs 8.34, P = 0.01).

**Conclusion:**

Patients with myalgia had lower virus load and those without myalgia had a higher virus load.

## Introduction

Though Chikungunya virus (CHIKV) was known since 1952, effective and timely management options are at various stages of development [1]. The virus is mainly transmitted by mosquitoes of *Aedes* genus (*aegypti*, *albopictus*) in the endemic regions and it has a short incubation period. Transmission of the disease is favoured by damp climatic conditions and more cases are usually registered during monsoon season. Increased global travel by humans also contributed to the wide spread of the disease [2].The advent of Kary Mullis invention, polymerase chain reaction(PCR) in 1985, revolutionized the diagnosis methods for various virus infections, including CHIKV [3]. Quantitative RT-PCR became a sensitive and potential tool to detect the RNA virus load during the acute phase of infection [4]. Studies investigating the relationship between virus load and the clinical features have been limited to animals and adults [1]. Children are one of the vulnerable groups to CHIKV infection. In spite of the disease being self-limiting, proper management at the right time can only reduce the complications [5]. Even though the mechanism of pathogenesis remains similar in both children and adults, symptoms might vary individually depending on age, immune response, virus load and cytokine levels, thus causing a change in the number of days of illness [6].

In virus detection laboratories, it is an established fact that antigen based tests (PCR) give positive results during viremic phase (1–4 days of infection) and antibody based tests give positive results seroconversion phase (after 5 days of infection) [7]. The symptomatic manifestation is the cumulative effect of virus replication, host response and the interactions between virus and host factors at various time points of infection period [8]**.** The variation in the amount of these individual components during different days of illness reflects in the form of varied symptoms at different time points.[9]. The targeted sites of replication of virus also vary with days of disease progression ranging from blood cells (macrophages, erythrocytes) to body cells (fibroblasts, synovial cells, muscle cells) [10]. Hence we should be equipped with specific antivirals that were effective for primary sites of CHIKV replication and candidates that target secondary sites of replication. Virus load patterns and their relationship to the development of severe dengue disease has been documented in certain studies [11]. In a resource-limited setting where virus load estimation is not possible, clinical features could be used as surrogate markers of virus load to guide treatment [12]. This present study collected data regarding CHIKV virus load, during the acute phase of infection in children and investigated the relationship between virus load and clinical manifestations of the disease.

## Materials and methods

### Subjects and samples

Between June 1, 2009, and May 31, 2010, we enrolled 338 patients (2 months to 18 years old) with suspected CHIKV symptoms during the acute phase of infection (day 1–4) at the outpatient department of Karnataka Institute of Medical Sciences (KIMS), Hubli. CHIKV symptoms were defined as the presence of fever (≥ 38°C) plus any symptoms like joint pain, rash, and myalgia and/or retro-orbital pain. Splenomegaly and hepatomegaly were detected and recorded after physical examination by the clinician following standard clinical methods. Patients who are already on any prescribed mode of treatment (allopathic, ayurvedic, homeopathy or unani) for present episodes, were excluded from this study.

### Sample processing and transport

Blood samples (3-5ml) were collected in sodium citrate vials. Plasma was separated by centrifugation, three aliquots were made for each sample and sent in dry ice to the department of pediatrics, All India Institute of Medical Sciences (AIIMS), New Delhi for testing. Once the samples were received (2009–2010), one aliquot was used for IgM testing and RNA extraction and the rest two aliquots were stored at -80°C for future use.

### RNA extraction and PCR

RNA was extracted using QIAamp Viral RNA Mini Kit as per manufacturer’s instructions. The extracted RNA was kept at -20°C and used for PCR testing within a week. Standard PCR with three sets of primers targeting E1 gene (930bp, 294bp, and 200bp) was used to confirm the CHIKV positivity before their inclusion for quantification. Dengue (DENV) PCR test for all four serotypes was performed to exclude dengue infected and co-infected samples (CHIKV & DENV) from this analysis.

### Real-time PCR

For quantitative RT-PCR, RNA extraction from plasma samples was performed using viral RNA extraction kit in accordance with the manufacturer’s instructions (QiAmp Viral RNA mini kit, Qiagen, Hilden Germany). Sybr green quantitative RT-PCR was used to detect CHIKV by targeting 200bp region of E1, envelope gene. SYBR Green is relatively cost effective and easy to use, technically based on binding the fluorescent dye to double-stranded deoxyribonucleic acid (dsDNA) and by optimization of SYBR Green method, its performance and quality could be comparable to other TaqMan method [13]. Forward primer 5’CAGCAAGAAAGGCAAGTGTGCG 3’, (10,959–10,980) (21b) and reverse primer 5’TGACTATGTGGTCCTTCGGAGGG3’, (11,136–11,158) (22b) were used. Reverse transcription was performed at 25°C for 10min, 37°C for 120 min and 85°C for 5 sec followed by 40 cycles of PCR (denaturation at 94°C for 15 sec, annealing at 54°C for 1 min, and extension at 72°C for 45 sec). Copy numbers in test samples were determined using a standard plot (lower limit of detection is 450 copies/ml) that was generated using in-house CHIKV RNA standards [14]. Confirmed CHIKV positive samples (n = 54) were taken for virus load estimation by SYBR green quantitative PCR method. Specific primers targeting 200bp region of E1 envelope gene of CHIKV were used for amplification. (Forward primer 5’-3’ CAGCAAGAAAGGCAAGTGTGCG, (10,959–10,980 (21b) and reverse primer 5’-3’ TGACTATGTGGTCCTTCGGAGGG, (11,136–11,158 (22b) and CHIKV RNA standards were prepared in house. Briefly, primers specific to 200bp region of E1 were designed to amplify the African CHIKV culture RNA. The forward primer used here was with T7 promoter sequence elongation (Forward **TAATACGACTCACTATAGGG**CAGCAAGAAAGGCAAGTGTGCG). The resulted PCR product was with T7 elongation and was gel purified. This purified product was used as the template for generating RNA using invitro transcription kit (MEGAscriptT7 Transcription Kit, Cat no:AM1333) following manufacturer’s instructions. The resulting RNA (sub genomic transcript) was quantified by nanodrop and concentration in nano grams was converted into copy numbers by using the formula and used as a standard after making serial dilutions. The whole experiment was performed on ABI7500 real-time machine. Briefly, RNA was extracted from 140ul of plasma samples using the QiAMp viral RNA mini kit (Qiagen, hilden germany). RNA was converted to cDNA by using high capacity cDNA reverse transcription kit (Applied Biosystems, Foster city, CA) according to manufacturer’s instructions and the cDNA was subjected to amplification of 200bp region of E1 gene in Sybr green real time PCR assay setup (ABI7000) using target specific primer set. Cycle threshold (ct) values obtained were plotted against the log dilutions of the RNA generated by Invitro transcription as described earlier [14] for the construction of the standard curve. The copy number in the plasma samples were calculated from the intersection points on the standard curve. Clinical symptoms of positive CHIKV were reviewed from case record forms (CRF’s) (attached in supplementary).

### Statistical analysis

Categorical variables were analyzed by chi-square/Fischer's exact test. P- Values less than 0.05 and near were considered to be statistically significant. Stata 12.0 was used for statistical analysis.

## Results

**Fig 1:**
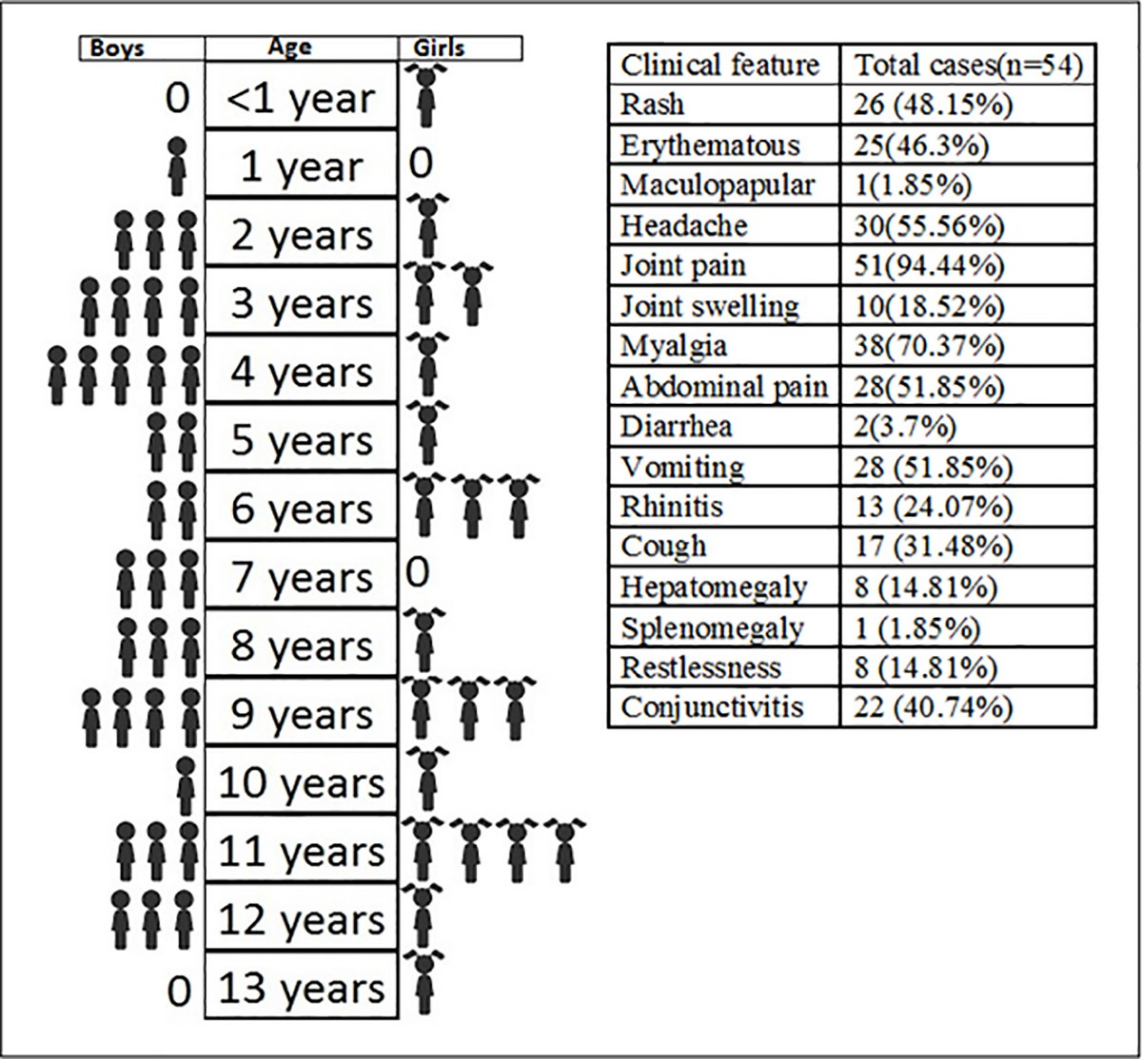
Total positive cases (n = 54) and percentage of observed clinical features in them.

**Clinical features:** Clinical features of patients with confirmed CHIKV infection (n = 54) were recorded. Among them, 20 were females and 34 were males. The age of the subjects ranged from 8 months to 13 years. All the patients presented with fever and other clinical symptoms. The symptoms that were frequently observed among the confirmed CHIKV cases include joint pain (94.44%), myalgia (70.37%), headache (55.56%), vomiting (51.85%) and abdominal pain (51.85%) (Fig 1). All the features were recorded during first 4 days of illness. Clinical features between two age groups were analyzed (Tables 1 and 2).

**Table 1:** Clinical features observed in two age groups.

	Total children (boys and girls) = 48		
Feature	Age 3–7 yrs (n = 23)	Age 8-13yrs (n = 25)	p-value*
Head ache	8	20	0.003
Joint Swelling	7	2	0.06
Hepatomegaly	6	0	0.008
Abdominal pain	17	9	0.01

*Fishers exact test was used to calculate the p values

Note: Children ≤2 yrs (n = 6) were excluded from this analysis, due to the possibility of misreporting of features. Other features where p-value is insignificant were not shown.

**Table 2:** Clinical features observed in two age groups of boys.

Clinical Feature	Young boys (Age: 3–7 years) (n = 16)	Older boys (Age: 8–13 years) (n = 14)	P-value*
Headache	5	11	0.014
Abdominal pain	11	4	0.066

*Fishers exact test was used to calculate the p values.

Note: Boys ≤2 yrs (n = 4) were excluded from this analysis, due to the possibility of misreporting of features. Other features where p-value is insignificant were not shown. In girls alone p-value was insignificant between two age groups.

### Virus load

Considering the day of fever onset as day 1, samples were collected during first 4 days of illness (day 1–4). The median copy number of CHIKV in 54 qRT-PCR positive patients was 1.3 X 10^8^ copies/ml (1.7x10^5^-9.9x10^9^copies/ml).

Observed clinical features were compared with estimated virus load. A significant difference in virus load was found between patients with and without myalgia (p = 0.01). The log mean virus load of the patients without myalgia is higher than in the patients with myalgia (8.34 vs 7.50). CHIKV load did not differ significantly in children with or without other clinical symptoms (Table 3).

**Table 3:** Association of virus load with the clinical features (n = 54).

Clinical feature		Virus load Mean±SD	P-value	Clinical feature		Virus load Mean±SD	P-value
Rash	No (28)	7.57 (1.15)	0.2154	Myalgia	No (16)	8.34 (0.880)	**0.0105**
	Yes (26)	7.95 (1.06)			Yes (38)	7.50 (1.123)	
Erythematous	No (29)	7.62 (1.16)	0.3367	Abdominal pain	No (26)	7.82 (1.12)	0.6704
	Yes (25)	7.91 (1.06)			Yes (28)	7.69 (1.12)	
Maculopapular	No (53)	7.73 (1.1)	-	Diarrhea	No (52)	7.75 (1.14)	0.7534
	Yes (1)	8.90 (-)			Yes (2)	8.00 (0.60)	
Headache	No (24)	7.73 (1.02)	0.8893	Cough	No (37)	7.75 (0.98)	0.9900
	Yes (30)	7.77 (1.20)			Yes (17)	7.75 (1.40)	
Joint pain	No (3)	8.04 (0.75)	0.6534	Hepatomegaly	No (46)	7.71 (1.08)	0.5120
	Yes (51)	7.73 (1.13)			Yes (8)	7.99 (1.34)	
Joint swelling	No (44)	7.75 (1.11)	0.9848	Splenomegaly	No (53)	7.73 (1.12)	-
	Yes (10)	7.76 (1.21)			Yes (1)	8.70 (-)	
Vomiting	No (26)	7.78 (1.22)	0.8554	Restlessness	No (46)	7.80 (1.07)	0.4898
	Yes (28)	7.72 (1.03)			Yes (8)	7.50 (1.41)	
Rhinitis	No (41)	7.76 (1.14)	0.9280	Conjunctivitis	No (32)	7.72 (1.13)	0.8066
	Yes (13)	7.73 (1.09)			Yes (22)	7.80 (1.11)	

Note: T test used to calculate p values. Shaded one is the significant p-value. Mean is the log mean of virus load.

## Discussion

CHIKV infection is now a global problem, as its spread is not limited to certain boundaries. Increased transport activities supported the transmission of the virus and number of cases are getting registered due to infected travellers all over the world [15,16]. Multicentre surveillance studies are the most effective way to estimate the magnitude of the disease burden at different geographic locations and to evaluate the transmission dynamics of Chikungunya infection [1,17]. Though Chikungunya is a self-limiting disease, severe manifestations like persistent arthralgia and myalgia may increase the duration of illness [18]. Dynamic transmission pattern of CHIKV and the morbidity associated with the disease has instigated many investigators not only to integrate the reports generated in different laboratories into the best possible sequence of events that can describe the disease progression during Chikungunya infection but also to develop vaccines, immunotherapeutic and chemotherapeutic molecules to combat the disease. Even though the cytokine/chemokine secretions start as soon as their producers receive stimulus from virus particles entry, which is the incubation period (first 4–7 days before the onset of symptoms), capturing the difference in their concentrations at this point is practically not possible as clinical symptoms are not manifested yet. There might be some common symptoms among CHIKV infected individuals like fever and joint pain but rest of the symptoms might vary depending on the difference in the host-virus interacting pattern, influenced by the immune response of the individual [19,20].

As observed in other studies involving other alphaviruses like Ross River virus (RRV), Sindbis virus (SINV), Mayaro virus (MAYV), O'nyong-nyong virus (ONNV), and Barmah Forest virus (BFV), virus load might have a direct influence on the expression levels of various cytokines which in turn might influence the presentation of various clinical symptoms [21–23]. Studies involving mice deficient in viperin showed an association between increased virus load and a more severe joint inflammation when compared to infected wild-type mice [24]. In a study conducted in aged rhesus monkeys, CHIKV infection resulted in higher and persistent virus replication due to defects in antiviral immunity [25]. Association between certain clinical features and cytokine induction during different phases of CHIKV infection was elucidated in certain studies [23,26,27] which provided a better understanding of the role mediated by immune mediators in CHIKV disease.

Joint pain was found to be the prominent feature among most of the study subjects (94.44%). Analysis of difference in the presentation of clinical symptoms between two age groups (3–7 yrs and 8–13 yrs.) showed that headache was present more in older children (8-13yrs) compared to the younger children (3–7 yrs.), (P = 0.003) (Table 1). Similar pattern was observed in young boys (n = 16) and older boys (n = 14) (P = 0.014) (Table 2). It emphasizes the fact that there might be a varied response to a virus infection between younger and older children. Further research on these lines could bring better insights into the relationship of host’s body makeup and the manifested clinical symptoms after a virus infection. However, a significant difference was not observed in the presentation of any clinical symptom between male and female groups.

**Fig 2:**
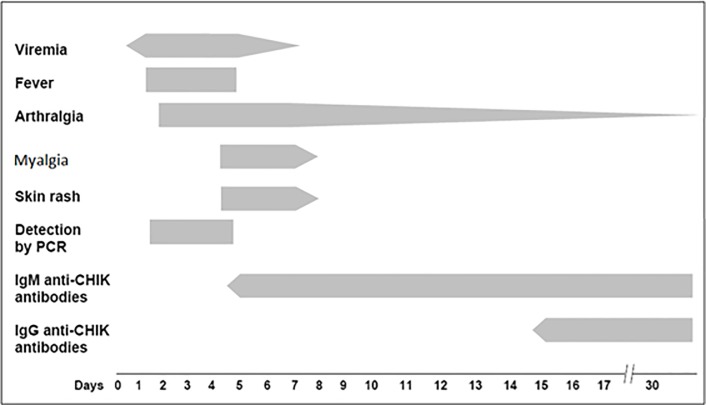
Temporal sequence of clinical symptoms and laboratory findings in chikungunya infection (re-presented after addition of myalgia from the article [8]).

Among all the recorded clinical features, only the presentation of myalgia varied with the difference in virus load (P = 0.0105). Interestingly, the subjects who don’t have myalgia had more virus load in them compared to subjects with myalgia. The translocation of virus particles towards myocytes coupled with host’s protective immune responses at that point of time might have some association with the reduction in virus load and manifestation of myalgia. Usually, viremia disappears after 4–5 days of infection, that’s one reason why antibody-based tests are used for detection after 5 days of infection [7]. Any feature manifested at this point of time can be due to the cumulative effect of virus load and the immune reaction against it. Pictorial depiction of temporal sequence of clinical features and laboratory findings in chikungunya infection in a review article clearly support this statement (Fig 2)[8]. The immune action (cytokines, antibodies) might cause lowering of the initial virus load with a simultaneous symptomatic presentation like myalgia. Recently, temporal analysis of CHIKV nsP3 within human cells, confirmed the continued presence of virus and cellular protein-complexes, thereby indicating their role in chronic infections due to persistence in various cell types, such as macrophages, muscle, and liver cells. (2018 reference). Chikungunya virus tropism to muscle cells was established in earlier studies [21,22,28]. A similar observation regarding myalgia and virus load was found in a study conducted on influenza virus [12]. All other variables like age, sex, socio-economic conditions, days of illness, co-infections might contribute their bit to the overall milieu of immune molecular levels and the subsequent presentation of clinical symptoms.

Limitations of the study were, 1) the association between virus load and days of illness was not clearly identified in our study because we did not have serial samples from the same patient. 2) Not estimating the role of cytokines and antibody levels in manifestation of clinical symptoms.

In our study we estimated virus load in 54 positive CHIKV patients by qRT-PCR and analysed it with presented clinical features (Table 3). Virus load had association with myalgia. Presence of myalgia was associated with lower viral load and higher viral load was associated with absence of myalgia. Presumably, myalgia can be an indicator of fading viremic phase, persistant virus replication inside host cells and raising seroconversion phase in patients. Insights about viremia levels, immune action, and clinical features can help in clinical decision making. Further studies involving estimation of cytokines, antibody levels and virus load in serial samples along with clinical features recording as continuous variables using mild-severity scales can help in deciphering the CHIKV pathogenesis, temporal sequence of manifestation of clinical features in humans in a more detailed way. This helps in stratifying treatment options for early and late phases of infection, by suggesting virus attachment and entry inhibitors that target structural proteins during first few days of infection i.e. viremic phase and virus replication inhibitors that target non-structural proteins during persistent virus replication phase.

## Supporting information

S1 Fig Case record form, page-1. (TIF)

S2 Fig Case record form, page-2. (TIF)

S3 Fig Patient consent form (English). (TIF)

S4 Fig Patient consent form (Hindi). (TIF)

S1 Table Raw data used for analysis. (XLSX)
