# Supplementary figures and images for: Virus load and clinical features during the acute phase of Chikungunya infection in children
Source: PLoS One. 2019 Feb 1;14(2):e0211036. doi: 10.1371/journal.pone.0211036 (PMC6358158; doi:10.1371/journal.pone.0211036)

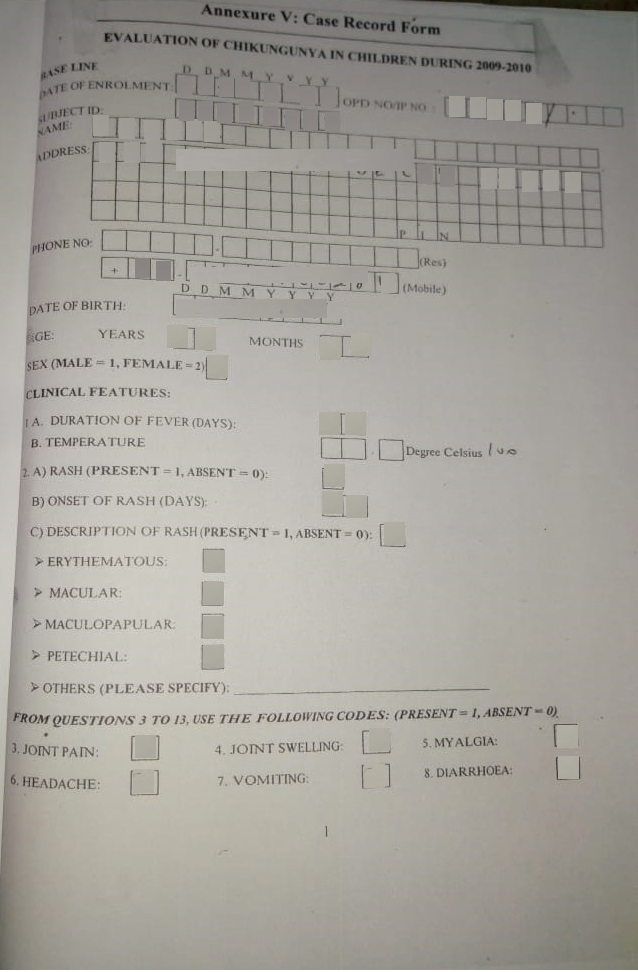

Supplement: S1 Fig — (TIF) [file pone.0211036.s001.tif]

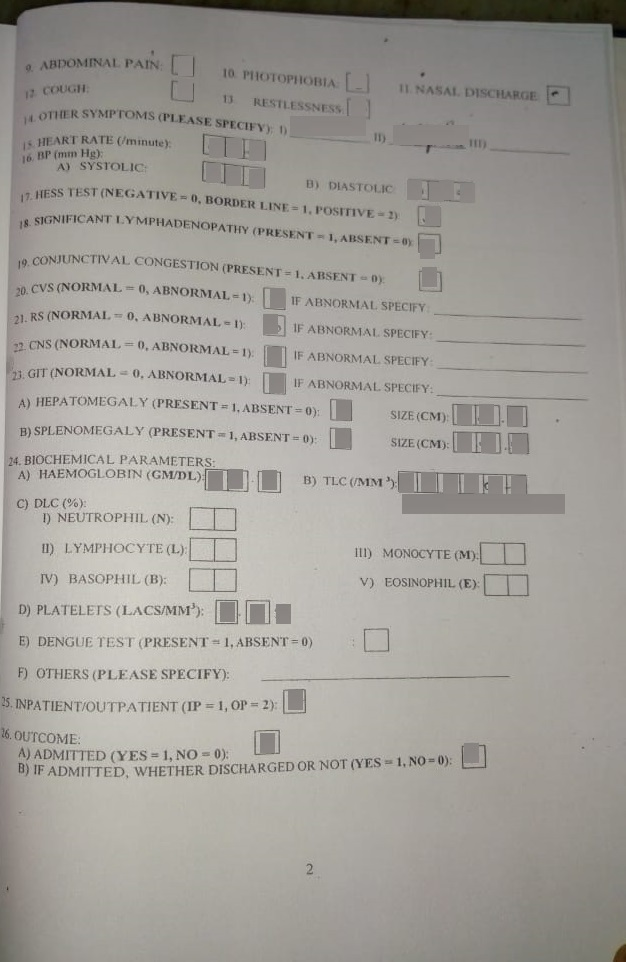

Supplement: S2 Fig — (TIF) [file pone.0211036.s002.tif]

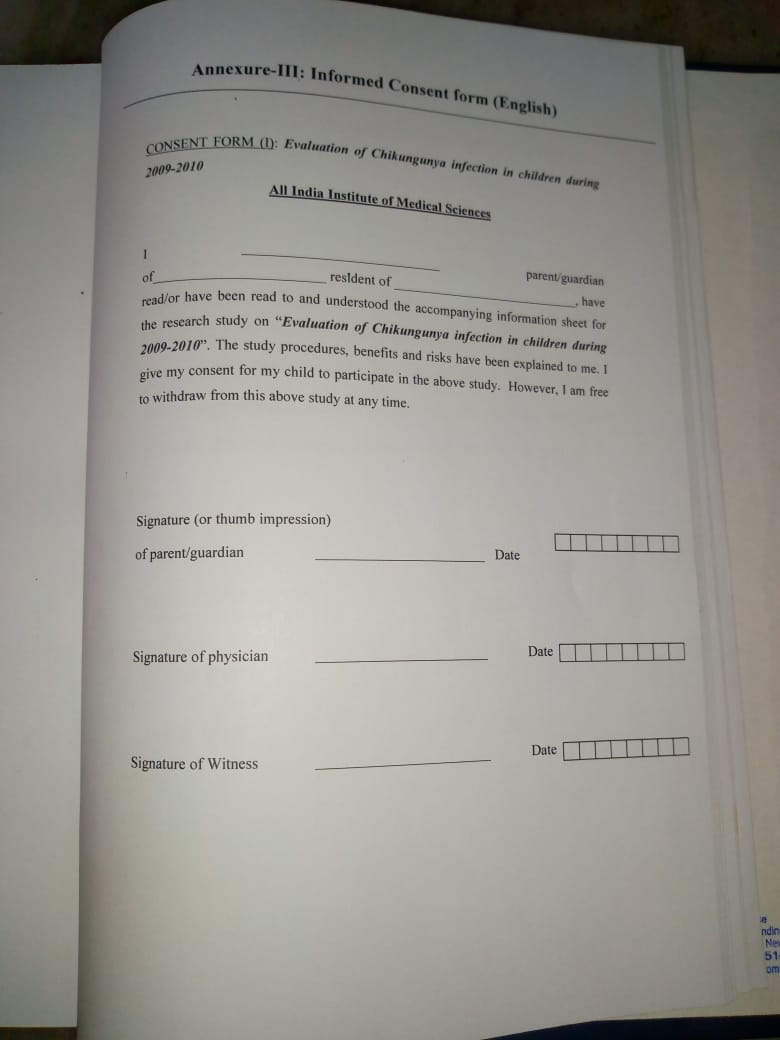

Supplement: S3 Fig — (TIF) [file pone.0211036.s003.tif]

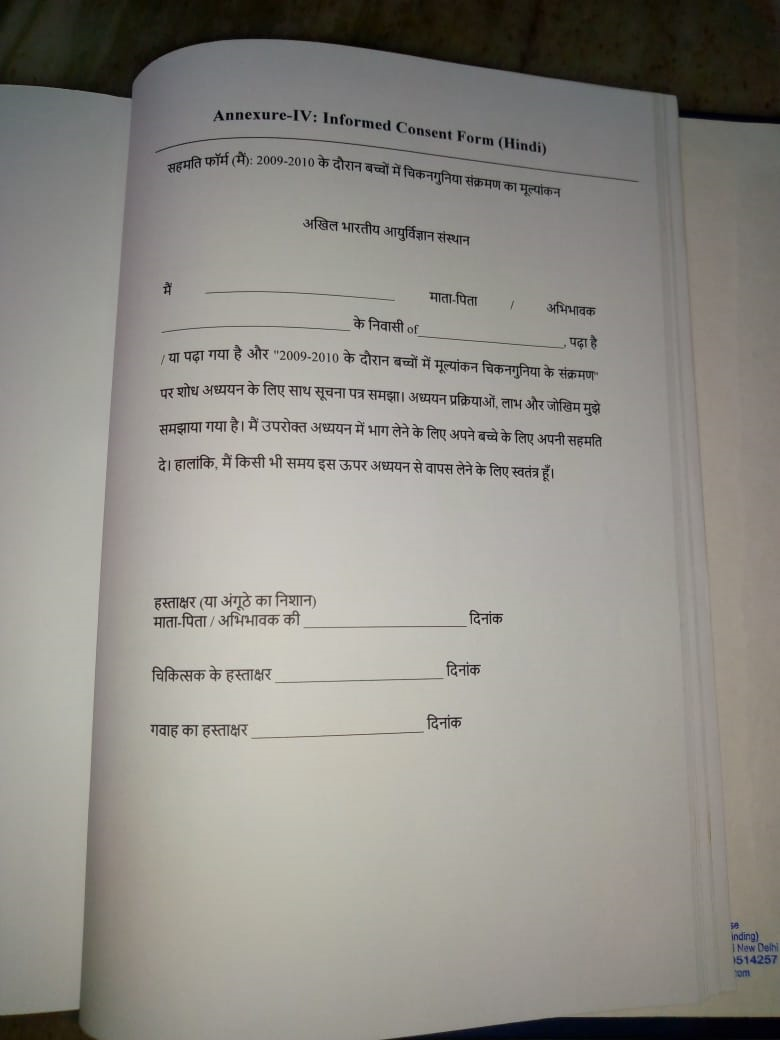

Supplement: S4 Fig — (TIF) [file pone.0211036.s004.tif]
